# Disposable Pencil Graphite Electrode for Diosmin Voltammetric Analysis

**DOI:** 10.3390/mi12040351

**Published:** 2021-03-25

**Authors:** Iulia Gabriela David, Alexandra-Gabriela Oancea, Mihaela Buleandră, Dana Elena Popa, Emilia Elena Iorgulescu, Adela Magdalena Ciobanu

**Affiliations:** 1 Department of Analytical Chemistry, Faculty of Chemistry, University of Bucharest, Panduri Av. 90-92, District 5, 050663 Bucharest, Romania; oancea.alexandra63@yahoo.com (A.-G.O.); elena.popa@chimie.unibuc.ro (D.E.P.); emilia-elena.iorgulescu@g.unibuc.ro (E.E.I.); 2 Discipline of Psychiatry, Neurosciences Department, Faculty of Medicine, “Carol Davila” University of Medicine and Pharmacy, Dionisie Lupu Street 37, 020021 Bucharest, Romania; adela.ciobanu@gmail.com; 3 Department of Psychiatry “Prof. Alexandru Obregia” Clinical Psychiatric Hospital, Berceni Av. 10, 041914 Bucharest, Romania

**Keywords:** diosmin, disposable working electrode, pencil graphite electrode, cyclic voltammetry, differential pulse voltammetry, adsorptive stripping voltammetry, dietary supplements

## Abstract

Diosmin (DIO) is a naturally occurring flavonoid with multiple beneficial effects on human health. The presence of different hydroxyl groups in diosmin structure enables its electrochemical investigation and quantification. This work presents, for the first time, diosmin voltammetric behavior and quantification on the cost-effective, disposable pencil graphite electrode (PGE). Diosmin oxidation on PGE involves two irreversible steps, generating products with reversible redox behaviors. All electrode processes are pH-dependent and predominantly adsorption-controlled. Differential pulse (DPV) and adsorptive stripping differential pulse (AdSDPV) voltammetric methods have been optimized for diosmin quantification o an H-type PGE, in 0.100 mol/L H_2_SO_4_. The linear ranges and limits of detection were for DPV 1.00 × 10^−6^–1.00 × 10^−5^ mol/L and 2.76 × 10^−7^ mol/L DIO for DPV and 1.00 × 10^−7^–2.50 × 10^−6^ mol/L and 7.42 × 10^−8^ mol/L DIO for AdSDPV, respectively. The DPV method was successfully applied for diosmin quantification in dietary supplement tablets. The percentage recovery was 99.87 ± 4.88%.

## 1. Introduction

**Figure 1:**
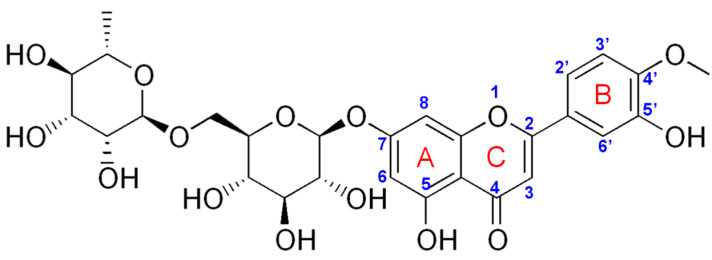
Chemical structure of diosmin (DIO).

Diosmin (3’,5,7-trihydroxy-4’-methoxyflavone 7-rhamnoglucoside) (DIO) (Figure 1), a natural flavone found in Rutaceae [1], commonly known as the citrus family, is easily obtained by hesperidin dehydrogenation. It was first isolated in 1925 from *Scrophularia nodosa*, and in 1969 it was introduced as a therapeutic agent [2]. After ingestion, diosmin is transformed into its aglycone form, diosmetin, and it has multiple beneficial effects on human health. This is due to the manifold biological and pharmacological activities possessed by diosmin, among which can be mentioned antioxidation, antidiabetes, anti-inflammation, anti-microorganism and anti-mutagenic activities, as well as neurological, cardiovascular, liver, retinal and renal protective effects [3]. Interesting and probably less known is the fact that DIO and its major metabolite, diosmetin, can be used in the treatment of Alzheimer’s disease (AD) [4] and as a memory restoring agent in the treatment of dementia [5]. DIO has beneficial effects on low neurological scores induced by brain damage on memory and long-term action potential by decreasing the tumor necrosis factor-alpha (TNF-α) proinflammatory cytokine production [3,6]. The clinical progression of AD is supported by the generation and accumulation of amyloid-(A) peptide. The inhibition of the glycogen synthase kinase-3 action by DIO and diosmetin has been shown to decrease the production of cerebral soluble Aβ and Aβ oligomer and tau hyperphosphorylation, resulting in the restoration of cognitive impairment in mice [4,7]. Elham et al. [8] established that any of diosmetin’s memory-enhancing properties in the chronic unpredictable mild stress condition may be linked to its ability to effectively regulate oxidative stress by increasing overall antioxidant potential and decreasing lipid peroxidation, while maintaining regular corticosterone levels. However, further research is required to determine the exact mechanism by which DIO, after its metabolization to diosmetin by the intestinal flora, causes memory deficits in chronic stress. Nowadays, the most popular and frequent uses for DIO are in the treatment of chronic vascular diseases and hemorrhoids. Thus, despite the poor solubility in water, diosmin, along with hesperidin, is present in various pharmaceutical formulations, food supplements or natural remedies that have phlebotonic and vascular-protecting properties against venous insufficiency [9].

The determination of diosmin along with other bioflavonoids is commonly performed by chromatographic [10,11,12], spectrophotometric [13,14,15] or fluorimetric [16] methods. Electroanalytical techniques are less exploited, although they have some economic advantages and convenient characteristics such as simplicity, speed and small amount of waste. Initial electrochemical studies considered determining diosmin using the bare carbon paste [17,18] and glassy carbon [19] electrodes. Chemical modification of the electrode surfaces led to broader linear ranges and improved detection limits [20,21]. The redox mechanism of some citroflavones, including diosmin, on the glassy carbon electrode surface was pointed out, highlighting the influence of methylation and glycosylation on the electrochemical processes [22]. Nevertheless, the analytical characteristics of the mentioned electrochemical methods can be improved, and better quantification methods for diosmin are still necessary and useful.

As was already mentioned, there are very few reports related to the voltammetric analysis of DIO, and none of them used a pencil graphite electrode (PGE). Pencil graphite leads are widely used as electrode material for electrochemical studies and quantification of various species [23,24,25,26]. PGEs have multiple advantages: uniform composition and surface of the leads, low background current, sensitivity, reproducibility, large and improvable active surface area, chemical inertness, good mechanical resistance and stability and large working potential ranges [27]. Unlike carbon and other conventional metal electrodes, PGEs can be easily miniaturized and modified. The sp^2^ hybridized carbon of graphite provides good analyte adsorption and higher conductivity. Moreover, in the case of the PGE, its active surface area can be adjusted by the proper selection of the lead length immersed in the analyzed solution, and the renewable surface is obtained by simply replacing the lead; meanwhile, there are tedious polishing procedures required for glassy carbon electrode (GCE) surface cleaning. Screen printed electrodes (SPCE) are far more expensive than the PGEs as disposable electrodes. Furthermore, in order to be re-used (no more than 20–30 times), the fouling of the SPCE surface necessitates cleaning of the electrode surface prior to each new electrochemical measurement [24,28]. The PGE is cost-effective, easy to use, readily commercially available, nontoxic and disposable by rapid replacement of the pencil lead. Therefore, the PGE is an economical, renewable and viable alternative tool to other expensive conventional electrodes, which have various drawbacks.

Taking into consideration the numerous significant activities possessed by DIO, its favorable effects on the cardiovascular and neurological systems, its recognized antioxidant activity and the fact that it is currently one of the most widely used natural compounds in the treatment of chronic venous insufficiency, the quantification of DIO is both useful and necessary. As one of the main ways of DIO uptake is the administration of pharmaceuticals or dietary supplements with DIO as an active principle, it is important to search for new simple and time- and cost-effective methods for its quantification in such samples. Hence, this work presents, for the first time, the electrochemical behavior of DIO on the unmodified PGE and, based on this, the development of simple and rapid differential pulse (DPV) and adsorptive stripping differential pulse (AdSDPV) voltammetric methods for its quantitative determination. The DPV method was successfully applied for DIO analysis in dietary supplements. The use of bare PGE represents another advantage in comparison with the modified electrodes, in which case additional steps and particular reagents are required for the preparation.

## 2. Materials and Methods

- Materials

Diosmin (DIO, >95%, analytical standard), NaOH (pellets), H_3_BO_3_ (1 g/tablet), acetic acid (≥97%, ACS reagent), H_3_PO_4_ (≥85 wt. % in water, ACS reagent), HCl (37%, ACS reagent), HNO_3_ (70%, ACS reagent) and H_2_SO_4_ (95.0–98.0%, ACS reagent) were provided by Sigma-Aldrich (Munich, Germany).

Diosmin-containing dietary supplement tablets (Revada; 600 mg DIO/tablet) produced by Weimer Pharma were purchased from a local natural products shop. Each tablet contained 600 mg diosmin, povidone, microcrystalline cellulose, corn starch and magnesium stearate.

The 1.00 × 10^−3^ mol/L DIO stock solution was prepared daily by dissolving the weight-appropriate amount of DIO in 2.00 mL 0.20 mol/L NaOH and subsequent dilution with deionized water to the mark of a 10.00 mL volumetric flask. When not in use, the stock solution was stored in the refrigerator. Working solutions were obtained just before the measurements by successive dilution with the corresponding supporting electrolyte.

For the investigation of the influence of the pH and nature of the supporting electrolyte on the voltammetric behavior of DIO, the universal Britton–Robinson buffer (BRB) with pH values in the range 1.81–9.15 and solutions of various acids at 0.10 mol/L, respectively, were employed.

All reagent solutions were prepared with deionized water.

- Instrumentation

A potentiostat/galvanostat Autolab PGSTAT 12 coupled to a PC running the GPES software and a 50.00 mL voltammetric cell was used for the electrochemical recordings. A Ag/AgCl (3.00 mol/L KCl) reference electrode, a Pt wire auxiliary electrode and a pencil graphite electrode (PGE), if not stated otherwise, were introduced in 10.00 mL of the analyzed solution contained in the voltammetric cell. The tested working electrodes were a platinum (Pt), a glassy carbon electrode (GCE) with a diameter of 0.30 cm (geometrical surface area 0.0711 cm^2^) and PGEs with graphite leads (from Rotring) of different hardness (2B, B, HB, H and 2H) and the diameter 0.05 cm. The PGE was obtained as previously reported [29,30,31] by introducing the graphite lead into a mechanical pencil holder so that 1.50 cm of the pencil mine remained outside. In each measurement, 1.00 cm of the graphite pencil lead was immersed in the investigated solution so that the geometrical surface area of the PGE was constant and equal to 0.1590 cm^2^. Clean and reproducible surfaces of the solid electrodes (Pt and GCE) were obtained by polishing the active electrode surface with alumina powder and then rinsing with deionized water and drying with filter paper.

The pHs of the tested solutions were measured with a combined pH-sensitive glass electrode connected to a Consort P901 Scientific Instrument pH/mV/°C-meter (Belgium).

- Procedures

DIO voltammetric behavior on PGE was investigated by cyclic voltammetry (CV) in the range 0.000 to 1.400 V. For quantitative assessment of DIO, the more sensitive differential pulse voltammetry (DPV) was applied in the potential range 0.000 to 1.500 V, with the following instrumental parameters: step potential 0.005 V, modulation amplitude 0.150 V, modulation time 0.05 s and interval time 0.10 s.

The standard addition method was used for the recovery studies and for the assessment of the DIO content in dietary supplement tablets (600 mg DIO/tablet).

Five tablets of the Revada dietary supplement with a claimed content of 0.6000 g DIO per tablet were accurately weighed and thoroughly mixed to a powder using a porcelain mortar and pestle. An appropriate mass of this powder, required to obtain 20.00 mL of 2.60 × 10^−4^ mol/L DIO solution, was dissolved in 2.00 mL 0.20 mol/L NaOH solution and 10.00 mL deionized water, and the suspension was filtered through a Whatman filter paper. The filter paper was rigorously washed with deionized water in order to ensure complete analyte recovery. The filtrate and the washing solutions were collected in a 20.00 mL volumetric flask and diluted to the mark with deionized water. Aliquots of this solution were 100-fold diluted with 0.10 mol/L H_2_SO_4_ to keep the sample concentrations within the linear range of the method and reduce the matrix effects [32]. Further, the samples were analyzed by DPV before and after each of the three successive additions of 0.30 mL of 5.26 × 10^−4^ mol/L DIO standard solution.

## 3. Results and Discussion

### 3.1. Optimization of the Working Conditions

The voltammetric behavior of an analyte and the sensitivity of its quantification are influenced by several chemical and instrumental parameters; therefore, these must be optimized during the method development steps.

#### 3.1.1. Selection of the Working Electrode

**Figure 2:**
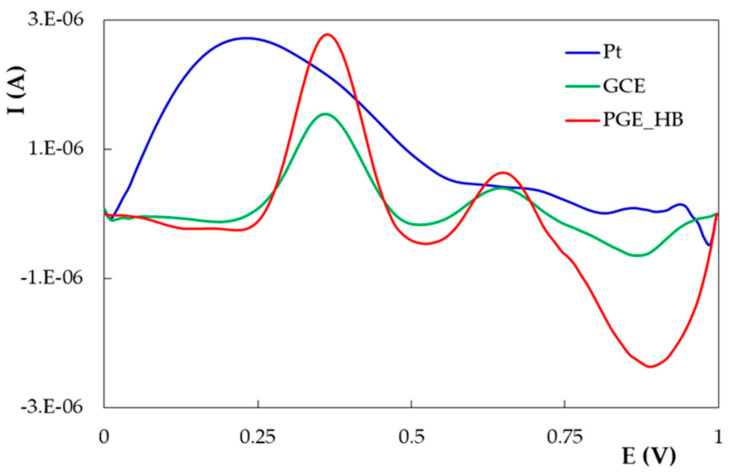
Differential pulse voltammograms recorded on different working electrodes for 1.89 × 10^−4^ mol/L DIO in 0.10 mol/L NaOH.

DIO voltammetric response was investigated by DPV on solid conventional working electrodes, i.e., Pt and GCE, and on a disposable PGE using an HB-type graphite pencil lead in NaOH as supporting electrolyte, because this is the medium in which DIO solubility is highest [33] due to the fact that in alkaline conditions DIO is ionized as a result of the dissociation of hydroxyl groups [34]. As can be observed in Figure 2, in an alkaline medium, DIO presented an intense and very broad signal on the Pt electrode, whereas two oxidation peaks were recorded on the carbon-based electrodes, and the one situated at a less positive potential (0.362 V) was higher. Despite the fact that the most intense anodic signal was observed at the HB-type PGE (PGE_HB), the sensitivities of the two carbon electrodes were similar (0.113 and 0.118 A × L/mol × cm^2^ for the PGE_HB and GCE, respectively). For further electrochemical investigations of DIO, the PGE was selected as the working electrode due to the following reasons: (i) it is disposable and its simple replacement is more rapid than the cleaning step of the solid electrode active surface and (ii) the electrode active surface area can be adjusted by the proper selection of the lead length immersed in the analyzed solution. The study started with the HB-type PGE because this kind of graphite pencil lead is well known and most commonly used. Graphite pencil leads are composite materials containing, besides graphite, a binder (clay or high polymer) and a lubricant (wax). Their hardness degree and notation according to the European Letter Scale (from 8H to 9B, through HB) depend on the graphite/binder mass ratio. The B (from blackness) type leads have more graphite and are softer, whereas the harder (H-type) composites have a higher binder content [27,35]. DIO oxidation signals were investigated by DPV on graphite pencil leads of different hardness (2B, B, HB, H and 2H) in both NaOH (due to DIO solubility in this medium, as mentioned above) and BRB pH 1.81 solutions because our previous investigations emphasized that many polyphenols present higher and better-defined anodic peaks in acidic media [29,30,31,36]. To characterize and compare the electrode materials, the sensitivity (S, expressed as A × L/mol × cm^2^) was used as a parameter independent of the electrode’s area, analyte concentration and supporting electrolyte. Despite the fact that all investigated PGEs were composite materials having the same constituents, the sensitivity of DIO oxidation response was different when using several graphite lead types. This fact can be attributed to the various graphite/high polymer ratios of the corresponding graphite leads. When NaOH was used as the supporting electrolyte, the sensitivity of the main oxidation signal of DIO was almost the same for leads of intermediate level of hardness (HB) and for the next hardest ones (H), which contain a higher proportion of polymer, whereas in acidic media (BRB pH 1.81), the H-type PGE clearly generated the most sensitive DIO oxidation signals in comparison to all the other investigated graphite leads of different hardness (Table 1). Therefore, as the highest sensitivities of the DIO anodic signals were recorded on the H-type PGE, in both acidic and alkaline media, PGE_H was selected as the working electrode for the next investigations.

#### 3.1.2. Selection of the Supporting Electrolyte

It is well known that the pH, the nature and the concentration of the supporting electrolyte influence the electrochemical behavior of an analyte, especially if the electrode processes also involve proton exchange reactions.

- The influence of the pH of the supporting electrolyte

The effect of the solution pH on the DIO voltammetric behavior on PGE_H was investigated using the universal BRB, in the pH range 1.81 to 9.15, by applying both the sensitive DPV and the CV (the voltammetric technique of choice for the study of analytes’ electrode processes).

**Figure 3:**
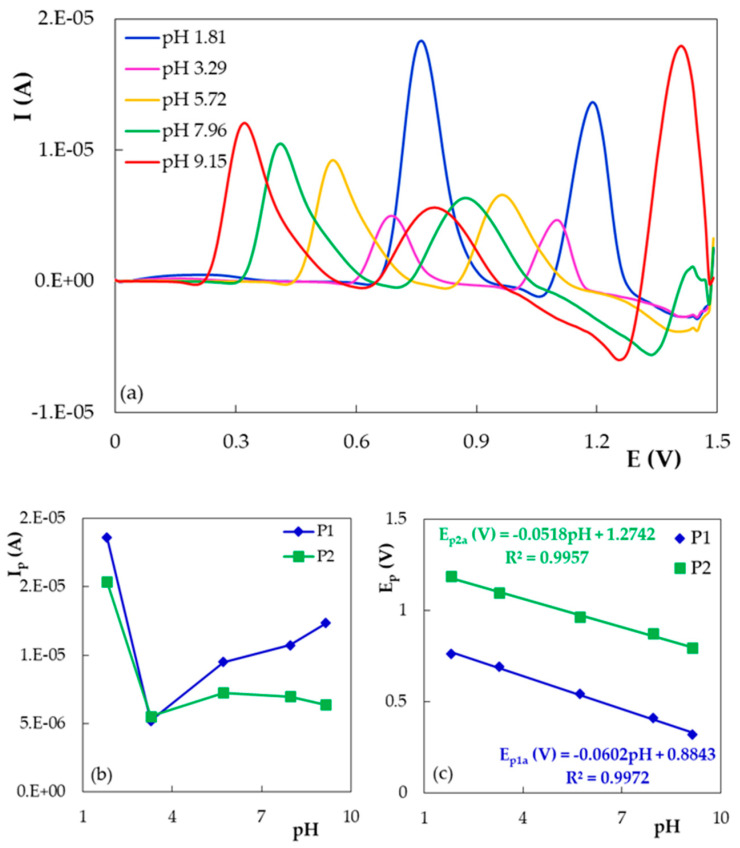
(**a**) Differential pulse voltammograms recorded on PGE_H for 5.00 × 10^−5^ mol/L DIO in BRB solution with different pH values. (**b**) The variation of the DIO DPV peak currents with the solution pH. (**c**) The DIO DPV peak potential dependencies on the solution pH.

On PGE_H, DIO presented two anodic DPV signals up to pH 9.00 and three peaks in more alkaline media (Figure 3a). This observation was in accordance with previous reports indicating that flavones are transformed into chalcone derivatives in NaOH solution [22]. By increasing the solution pH, the signals decreased until pH 3.29 and increased at higher pHs (Figure 3b), whereas their peak potentials shifted towards less positive values, indicating that the oxidation processes were accompanied by proton exchanges. Comparing the slope values of the linear E_p_ = f(pH) dependencies (Figure 3c) with the theoretical one of 0.05916x/n, according to the Nernst equation, where x and n represent the number of exchanged protons and the number of exchanged electrons, respectively, one can conclude that equal numbers of protons and electrons are involved in both DIO oxidation reactions.

**Figure 4:**
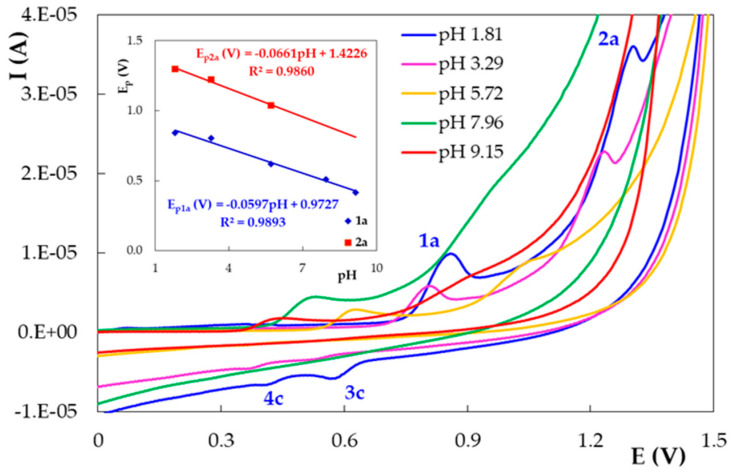
First scan of the cyclic voltammograms recorded on PGE_H for 1.00 × 10^−5^ mol/L DIO in BRB solution with different pH values; v = 0.100 V/s. The inset shows the peak potential dependencies vs. the solution pH.

**Figure 5:**
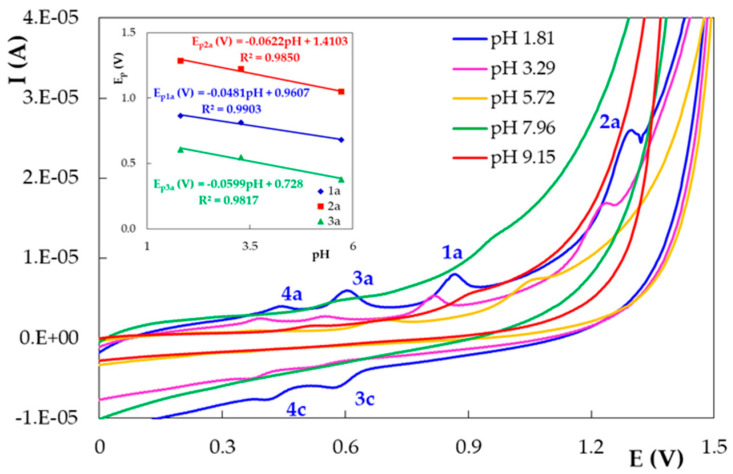
Second scan of the cyclic voltammograms recorded on PGE_H for 1.00 × 10^−5^ mol/L DIO in BRB solution with different pH values; v = 0.100 V/s. The inset shows the peak potential dependencies vs. the solution pH.

In the first scan, DIO cyclic voltammograms recorded on PGE_H showed two anodic peaks (Figure 4). The signal at less positive potentials (with E_p1a_ between 0.843 V at pH 1.81 and 0.415 V at pH 9.15) was observed on the entire investigated pH range, whereas a second oxidation signal situated at potentials over 1.000 V (with E_p2a_ between 1.300 V at pH 1.81 and 1.000 V at pH 5.72) appeared only at pH values up to 5.72. The peak potentials of these two signals shifted linearly towards more positive values when the pH values decreased (Figure 4 inset). At the reversed scan of the first potential cycle, DIO cyclic voltammograms presented only in acidic media (pH 1.81 and 3.29) two cathodic signals situated at potentials (E_p3c_ ~0.500–0.600 V and E_p4c_ ~0.350–0.400 V) far away from those corresponding to the previously discussed anodic signals. Thus, one can assume that DIO was irreversibly oxidized on PGE_H and the cathodic peaks were due to the reduction of DIO oxidation products [21]. This assumption was supported by the fact that in the direct scan of the second potential cycle, for pH values up to 5.72, two more anodic signals were observed (E_p3a_ ~0.600 V and E_p4a_ ~0.400 V) in addition to those already emphasized during the first scan (Figure 5). Due to the small differences between the potential values of peaks 3a and 3c, as well as 4a and 4c, and the corresponding I_pa_/I_pc_ values being slightly higher than 1.00, these peaks can be attributed to two quasireversible redox couples, corresponding to the DIO oxidation products previously formed, namely *ortho*- and *para*-quinone, which are reduced in two parallel processes to catechol (peak 3c) and hydroquinone (peak 4c), respectively. The absence of the peak couples 3a–3c and 4a–4c at pH values above 5.72 can be explained by the fact that the catechol and hydroquinone reversible redox behavior is prevalent in an acidic environment. In alkaline media, the oxidation of the deprotonated –OH groups can involve irreversible bond breaking and/or dimerization and results in the decrease in anodic signal intensity and the absence of the cathodic peaks [37].

The peak potential values of the signals observed in the second potential cycle decreased with the increase in pH, suggesting that the corresponding electrode reactions also involved protons. It must be specified that peaks 3a, 1a and 2a appeared at pH values up to 5.72, whereas the signals 3c, 4a and 4c could be observed only in acidic media (pH 1.81 and 3.29, respectively) (Figure 5 inset). According to the slope values of the E_p_ = f(pH) plots for the DIO cyclic voltammetric peaks, all the redox processes generating them involved an equal number of protons and electrons, a fact demonstrated also by DPV for the main anodic peaks (1a and 2a).

This very complex electrochemical behavior of DIO on PGE as a function of the solution pH is the result of the ionizable electroactive groups contained in its molecule. Depending on the pH in the solution, various protonated, neutral or ionized DIO species with different electrochemical characteristics may (co)exist, and their concentrations will vary with the pH.

- The influence of the nature of the supporting electrolyte

**Figure 6:**
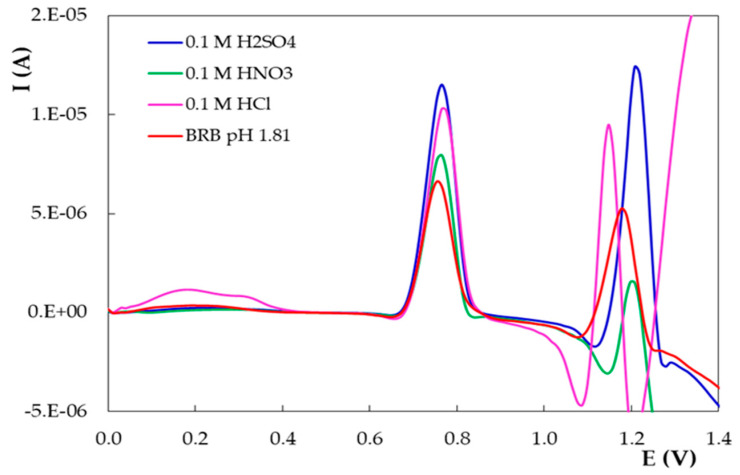
Differential pulse voltammograms recorded on PGE_H for 1.00 × 10^−5^ mol/L DIO in different acidic supporting electrolytes.

As the above-discussed results revealed that the highest main oxidation signal (1a) of DIO on PGE_H (Figure 3b, Figure 4 and Figure 5) was recorded in a solution of pH 1.81, the effect of some acidic supporting electrolytes on the DPV response of DIO was further tested (Figure 6). The results indicated that the most sensitive DIO anodic signals were obtained in 0.10 mol/L H_2_SO_4_; therefore, this medium was used as the supporting electrolyte in the next investigations.

#### 3.1.3. The Stability of the DIO Solution

**Figure 7:**
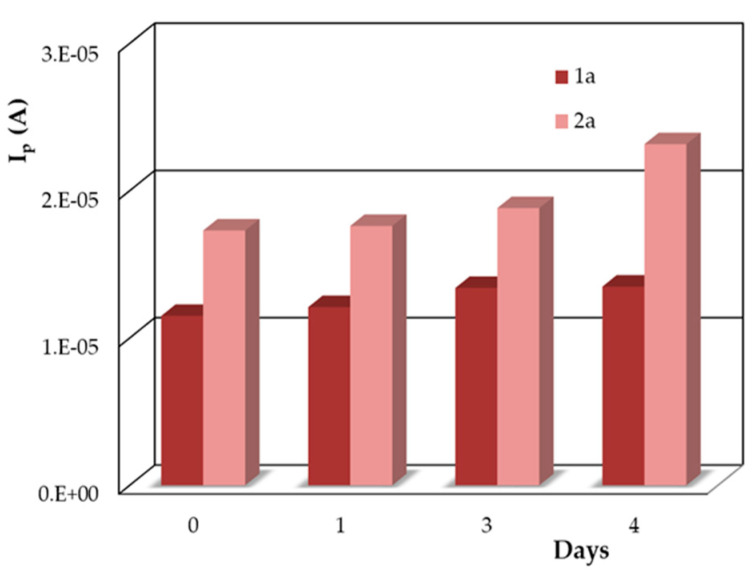
DPV anodic peak currents recorded on PGE_H for 1.00 × 10^−5^ mol/L DIO in 0.10 mol/L H_2_SO_4_ obtained on different days from the same stock solution (1.00 × 10^−3^ mol/L DIO in 0.04 mol/L NaOH).

It is well known that antioxidant solutions are not very stable; hence, the stability of the DIO stock solution was tested by monitoring the voltammetric response of the working solutions freshly prepared from the same stock solution after several days of storage in the refrigerator (Figure 7). The increase in both DIO anodic peak currents (1a and 2a) indicated that some transformations occurred in the solution; therefore, the stock solution must be prepared daily.

Regarding the stability of the working solutions, it was observed that in the acidic medium used for the electrochemical analysis, they were stable for at least one hour if the DIO concentration was up to 1.00 × 10^−5^ mol/L, whereas the 5.00 × 10^−5^ mol/L DIO working solutions were stable for about 10 min, enough time for performing at least five voltammetric recordings on the same solution.

However, the low stability of the analyte solution did not influence the results of the sample analysis because (i) both the standard DIO stock solution and the solutions of the investigated samples were prepared just before the analysis and used within the same day and (ii) the diluted sample solutions without and after the additions of DIO stock solution had concentrations lower than 1.00 × 10^−5^ mol/L and stayed in the voltammetric cell for less than one hour (about 40–45 min).

### 3.2. Voltammetric Behavior of DIO on the Pencil Graphite Electrode (PGE_H)

**Figure 8:**
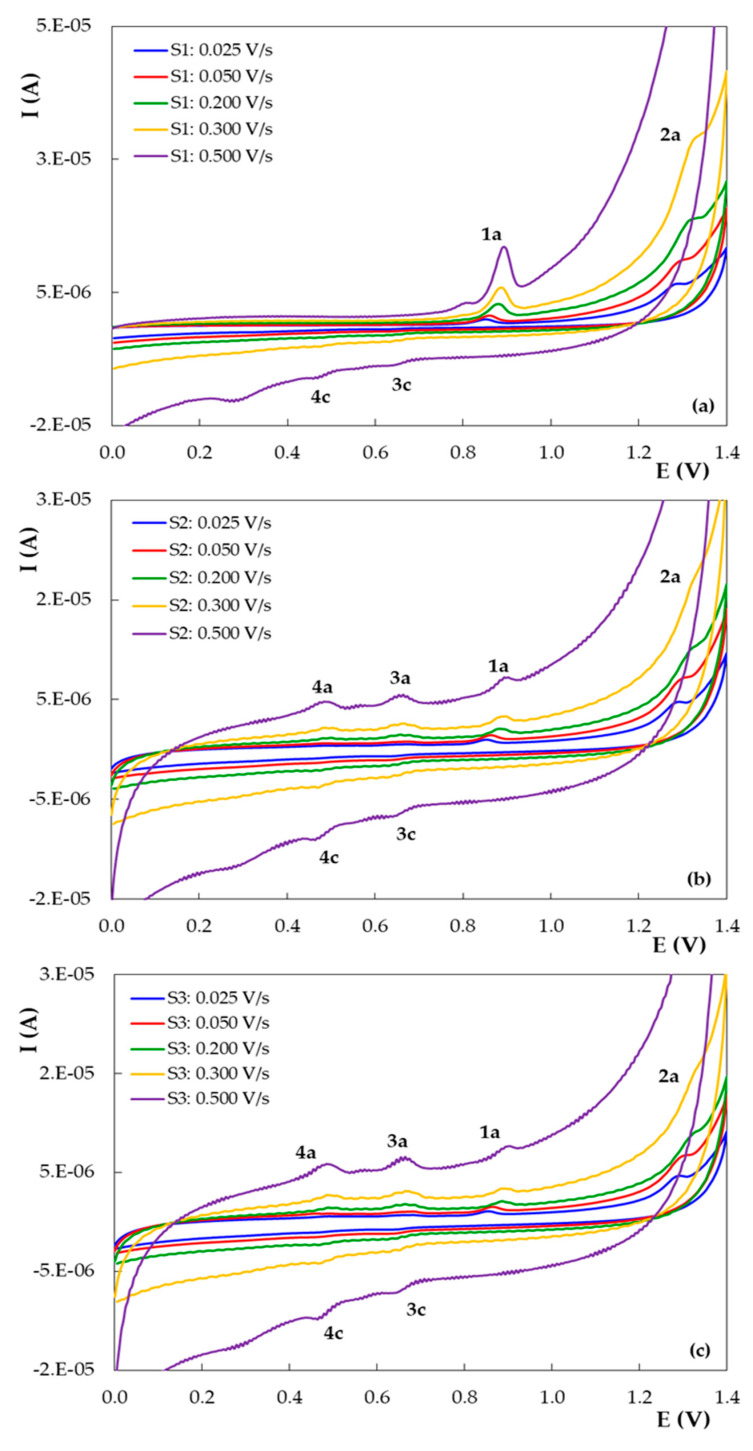
Cyclic voltammograms recorded at different scan rates on PGE_H for 1.00 × 10^−5^ mol/L DIO in 0.10 mol/L H_2_SO_4_: (**a**) first scan; (**b**) second scan; (**c**) third scan.

The voltammetric behavior of DIO on PGE_H was investigated in 0.10 mol/L H_2_SO_4_ by recording the first three potential scans at different sweep rates (Figure 8). In the first voltammetric cycle (Figure 8a) DIO showed in the direct scan a well-defined anodic signal (1a) with peak potentials situated between 0.835 and 0.894 V and a less pronounced wave (2a) at more positive potentials (1.274 to 1.343 V). In the negative-going scan, a reduction signal (3c) appeared at about 0.600 V. The second cathodic wave (4c) at approximately 0.450 V was observed only at very high scan rates (more 0.300 V/s). In the next two successive voltammetric cycles (Figure 8b,c) recorded in the same solution, on the same working electrode, one can notice, in the positive-going scan, beside the peaks (1a and 2a) also existing in the first CV scan, two more anodic signals: 4a with E_p4a_ of ~0.480 V and 3a having an E_p3a_ of ~0.680 V. When reversing the sweep direction towards reduction potentials, two small cathodic waves occurred, namely 4c with E_p4c_ of ~0.430 V and 3c with E_p3c_ of 0.635 V, which correspond to the anodic peaks 4a and 3a, respectively. The peak current ratios of the signals couples 3a–3c and 4a–4c were slightly higher than 1, for all scans, leading to the conclusion that they correspond to the quasireversible redox reactions of DIO oxidation products. Considering the results of previous studies regarding the voltammetric behavior of DIO and related compounds at GCE [22], peak 1a is due to the oxidation of the 5’-OH from the B ring resulting in *ortho*-quinone and *para*-quinone type oxidation products, which are further involved in quasireversible redox reactions generating peaks 3a–3c and 4a–4c, respectively, whereas peak 2a was attributed to the electrooxidation of the 5-OH group (ring A).

**Figure 9:**
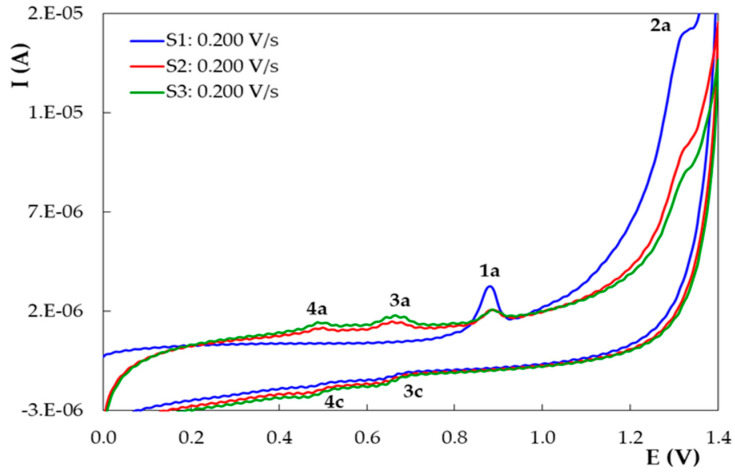
Successive repetitive cyclic voltammograms recorded on PGE_H for 1.00 × 10^−5^ mol/L DIO in in 0.10 mol/LH_2_SO_4_; v = 0.200 V/s.

The decrease in DIO anodic peaks 1a and 2a and the increase in the peaks corresponding to the redox couples 3 and 4 (peaks 3a–3c and 4a–4c, respectively) (Figure 9) upon successive CVs suggested that DIO oxidation products were adsorbed on the PGE_H surface. This assumption is supported by the fact that the oxidation reactions of monophenols also include the formation of polymers which may passivate the electrode surface [37].

A mechanism for the reactions generating peaks 1a, 3a and 3c was suggested by Li et al. [21], but there is no literature information regarding peaks 2a, 4a and 4c. However, in order to establish a mechanism for the processes generating these signals, more detailed studies using complementary methods (e.g., coulometry) are necessary.

The currents of all the other five signals recorded in the three potential scans increased with the scan rates from 0.025 to 0.300 V/s. From the regression equations of the different dependencies of the peak currents on the scan rate (Table 2), one can conclude that all voltammetric signals observed for DIO on PGE_H are mixed, predominantly adsorption-controlled electrode processes.

### 3.3. The Development and Validation of a Voltammetric Method for DIO Quantification with the Pencil Graphite Electrode 

#### 3.3.1. Linearity and Limits of Detection and Quantification

**Figure 10:**
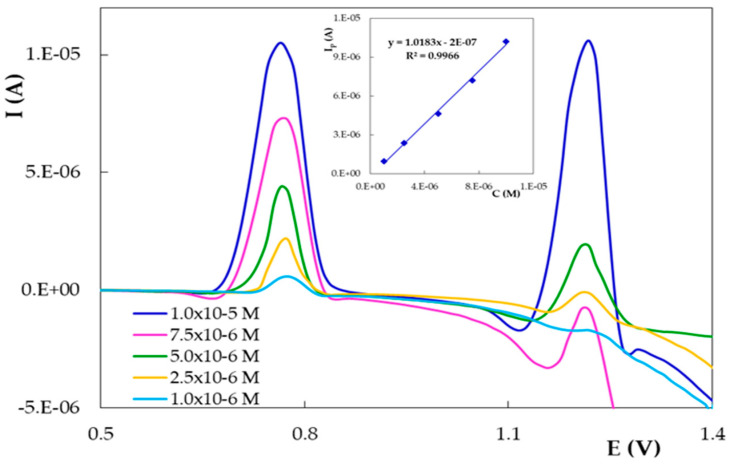
Differential pulse voltammograms recorded on PGE_H for different concentrations of DIO in 0.10 mol/L H_2_SO_4_. The inset shows the corresponding calibration curve.

For the quantitative voltammetric determination of DIO on the disposable electrode, the more sensitive DPV method was applied using the previously optimized working conditions (PGE_H and 0.10 mol/L H_2_SO_4_). The influence of the analyte concentration on DIO oxidation peaks was investigated in the range 1.00 × 10^−7^ to 2.50 × 10^−5^ mol/LDIO. As can be observed from Figure 10, both DIO anodic signals were enhanced by the concentration increase from 1.00 × 10^−6^ to 1.00 × 10^−5^ mol/L DIO, but for further quantitative investigations, only the peak situated at ~0.780 V was exploited due to the fact that it is not affected by the background current. The current of this peak varied linearly with DIO concentration (Figure 10 inset).

**Figure 11:**
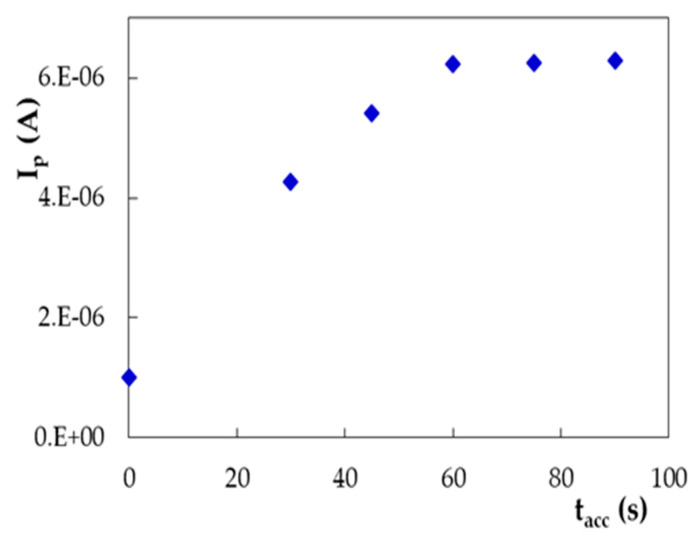
The influence of the accumulation time (t_acc_) on DIO DPV oxidation peak current recorded on PGE_H for 1.00 × 10^−6^ mol/L DIO in 0.10 mol/L H_2_SO_4_; E_acc_ = 0.000 V.

The linear range of one order of magnitude (1.00 × 10^−6^ to 1.00 × 10^−5^ mol/L DIO) obtained by DPV on PGE_H is sufficient for DIO quantification in dietary supplements, but for the analysis of real samples, e.g., citrus fruit parts and biological fluids, a more sensitive method is necessary. Therefore, the DIO adsorption properties on PGE, deduced from the previous CV studies, were investigated in order to exploit them for a more sensitive stripping voltammetric analysis. Consequently, the influence of the accumulation time and potential on DIO oxidation peak from about ~0.780 V was examined. DP voltammograms recorded after maintaining the PGE-H at 0.000 V in a 1 × 10^−6^ mol/L DIO solution (the lowest concentration of the linear range obtained by DPV) for different times (0–90 s) emphasized that the peak current increased with the accumulation time up to 60 s and remained constant for longer deposition periods (Figure 11) due to the saturation of the electrode surface with the analyte molecules.

**Figure 12:**
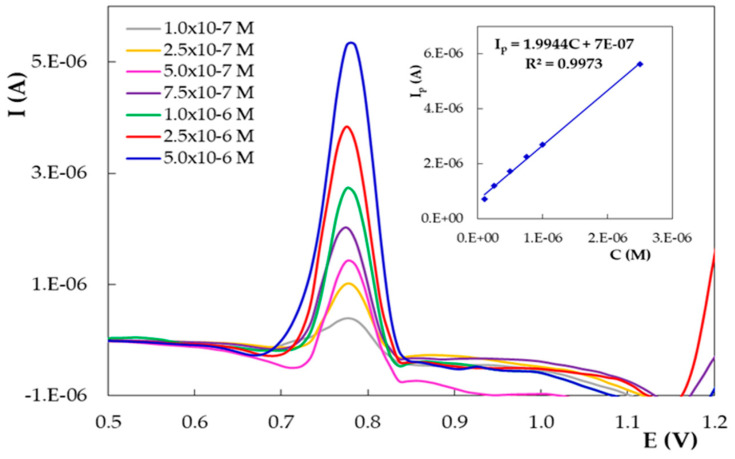
Adsorptive stripping differential pulse voltammograms recorded on PGE_H for different concentrations of DIO in 0.10 mol/L H_2_SO_4_; E_acc_ = 0.000 V and t_acc_ = 60 s. The inset shows the corresponding calibration curve.

The effect of the accumulation potential (E_acc_) on the same DIO oxidation peak was investigated by varying this parameter in the range −0.200 to 0.200 V, while t_acc_ was kept constant at 60 s. The DIO anodic signal did not change with the potential applied to the PGE_H during the deposition step due to the non-electrochemical nature of the DIO adsorption process on the electrode surface. Thus, the influence of the concentration on the main oxidation signal of DIO from ~0.780 V was investigated between 1.00 × 10^−7^ and 1.00 × 10^−5^ mol/L DIO by adsorptive stripping differential pulse voltammetry (AdSDPV) on PGE_H carried out applying the optimized deposition parameters, i.e., E_acc_ 0.000 V and t_acc_ of 60 s (Figure 12). The accumulation step was done under stirring conditions and the recordings were performed in still solution, after an equilibration time of 5 s. The obtained peak currents varied linearly with the analyte concentration between 1.00 × 10^−7^ and 2.50 × 10^−6^ mol/L DIO (Figure 12 inset). At DIO concentrations higher than 2.50 × 10^−6^ mol/L, the calibration curve was flattened due to the saturation of the electrode surface with adsorbed DIO molecules.

The limits of detection (LoD) and quantification (LoQ) were calculated as 3.3 s_x/y_/b and 10 s_x/y_/b, respectively where s_x/y_ is the residual standard deviation and b is the slope of the calibration curve [38]. The LoQs of the DPV and AdSDPV on PGE_H methods developed for the quantitative determination of DIO, using the oxidation signal from ~0.780 V were 8.35 × 10^−7^ and 2.47 × 10^−7^ mol/L DIO, respectively. The linear ranges and the LoDs of these methods are listed in Table 3. As already mentioned in the introduction, there are very few literature reports on the voltammetric analysis of DIO, and these are summarized in Table 3. As can be seen, the methods previously published have better performance characteristics in comparison to those presented in this study, but the newly described methods also have good performances along with the following main advantages: (i) they use a cheap and commonly available unmodified disposable electrode, (ii) they do not need expensive and toxic materials and (iii) they are rapid and easy.

#### 3.3.2. Repeatability

The method precision for DIO voltammetric determination on PGE_H, expressed as percentage relative standard deviation (RSD%), was evaluated at three concentration levels as repeatability (intraday assay) and intermediate precision (interday assay—three successive days). Thus, 20 voltammograms at each of the three different DIO concentrations (lowest and highest concentrations of the linear range and an intermediate concentration) were recorded. Each measurement was performed on a new pencil lead. All obtained RSD% values (Table 4) were within the accepted limits for the corresponding concentrations [39].

#### 3.3.3. Selectivity Studies

There is a huge variety of natural polyphenols, and their electrochemical detection is usually based on the oxidation processes of the hydroxyl groups grafted on the aromatic rings. However, depending on the molecular structure and on the other existing substituents, the oxidation peak(s) can be situated at different potentials. The oxidation signal obtained on a PGE in 0.10 mol/L H_2_SO_4_ for caffeic acid [29], rosmarinic acid [30] and chlorogenic acid [31] was situated at around 0.550 V, whereas, in the same conditions, naringenin [36] and hesperidin, which are structurally related to DIO, exhibited the main anodic peak at potentials similar to DIO, interfering with its voltammetric determination. Therefore, the developed DPV on PGE method is not selective enough to enable the separate quantification of structurally related polyphenolic antioxidants, but based on the “electrochemical index” defined by Blasco et al. [40], it is able to discriminate between polyphenols with different antioxidant powers (AOPs) as follows: polyphenols which have peaks at potentials lower than 0.300 V at pH ~7.00, like the above-mentioned hydroxycinnamic acids, are considered to have a high AOP, whereas the DIO-related flavonoids with peak potentials of ~0.550 V at pH ~7.00 are classified as compounds with intermediate AOP. Thus, when applied to samples with complex matrices, the method described in this work can only determine the total content of polyphenols with intermediate AOP, expressed as milligram equivalent diosmin per milliliter or per gram sample. Consequently, the objective of this study consisted of developing a reliable voltammetric method for DIO determination in pharmaceutical formulations containing only this active principle.

#### 3.3.4. Recovery Studies and Analytical Applications

The practical applicability of the voltammetric determination of DIO on PGE_H was tested by analyzing the DIO content of dietary supplements using DPV, due to the fact that the linear range of the developed method was satisfactory for DIO quantification in this type of sample. The standard addition method was employed for recovery studies and the estimation of DIO content in Revada (600 mg diosmin, Weimer Pharma) tablets, commercially available in local drugstores. The samples were prepared and analyzed according to the procedures described in Section 2. 

**Figure 13:**
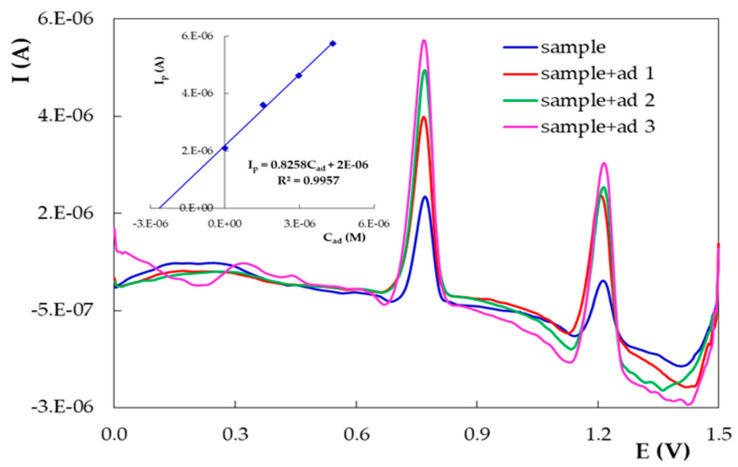
Differential pulse voltammograms recorded on PGE_H for 10 mL sample solution in 0.10 mol/L H_2_SO_4_ before and after each addition of 0.30 mL of 5.26 × 10^−4^ mol/L DIO standard. The inset shows the corresponding calibration curve.

Five replicate samples were analyzed. For each measurement, a new PGE_H was used. The differential pulse voltammograms recorded for the tablet solutions diluted with 0.10 mol/L H_2_SO_4_ presented only the characteristic DIO oxidation peaks at ~0.780 and ~1.200 V (Figure 13), indicating the absence of any interference from the ingredients of the tablets. The peak currents linearly increased with the addition of DIO stock solution (Figure 13 inset), thus enabling the quantitative determination of the analyte. For further investigations, only the peak situated at less positive potentials (~0.780 V) was exploited, because this is the main oxidation signal of DIO, and the influence of concentration on DIO voltammetric signals was also investigated using only this signal. The DPV peak currents recorded on PGE_H for the sample in 0.10 mol/L H_2_SO_4_, before and after each standard addition of the DIO stock solution, were measured and used to assess the DIO content of the Revada tablets and the percentage recovery values (%R) (Table 5), considering all the dilutions carried out during the sample preparation procedure. The results emphasized a very small difference between the DIO content determined by the developed method and that claimed by the producer.

## 4. Conclusions

This work presents, for the first time, the complex voltammetric behavior of DIO on a disposable composite graphite electrode, namely the pencil graphite electrode (PGE). DIO is irreversibly oxidized on PGE, generating products that are reversibly reduced in the subsequent negative-going scan. The electrode processes were pH-dependent and predominantly adsorption controlled. The main oxidation signal of DIO, situated at ~0.780 V (in 0.10 mol/L H_2_SO_4_ medium), was exploited for the development of DPV and AdSDPV methods for DIO quantification. Despite the fact that the voltammetric methods previously reported for DIO determination have better performance characteristics, they used modified electrodes, whereas the ones described here employed a bare, unmodified PGE. Thereby, the analysis is simple (no need to modify the electrode surface) and fast (no time is consumed for electrode surface cleaning) as well as user- and eco-friendly because the working electrode consists of the well-known nontoxic graphite pencil leads, which are easily commercially available worldwide at very low prices (approximately $0.13) [31]. Hence, the developed DPV method was used with good results for the rapid and cost-effective determination of DIO in dietary supplements.

## Figures and Tables

**Table 1:** Peak potentials and sensitivities of DIO oxidation signals obtained by DPV on PGEs with different hardnesses.

PGE Type	E_p1_ (V)	S_1_ (A × L/mol × cm^2^)	E_p2_ (V)	S_2_ (A × L/mol × cm^2^)
		0.10 mol/L NaOH		
2H	0.353	0.077	0.645	0.030
H	0.353	0.110	0.645	0.065
HB	0.363	0.113	0.655	0.028
B	0.383	0.065	0.655	0.024
2B	0.383	0.049	0.655	0.026
		BRB pH 1.81		
2H	0.755	0.331	1.178	0.279
H	0.775	0.343	1.178	0.337
HB	0.755	0.182	1.178	0.095
B	0.785	0.130	1.178	0.096
2B	0.765	0.120	1.178	0.153

**Table 2:** The parameters of the regression equations corresponding to the different dependencies of the peak currents on the scan rate for DIO oxidation signals obtained by the CVs from Figure 8.

Peak/Scan ^1^	I_p_ = f(v)			I_p_ = f(v^1/2^)	logI_p_ = f(logv)		
	Slope	Intercept	R^2^		Slope	Intercept	R^2^
1a/1	1 × 10^−5^	3 × 10^−7^	0.9889	Non linear	0.7669	−5.0028	0.9861
2a/1	3 × 10^−6^	2 × 10^−7^	0.9974	Non linear	0.6662	−5.5863	0.9999
3c/1	9 × 10^−7^	2 × 10^−8^	0.9818	Non linear	0.7747	−6.1465	0.9826
3a/2	2 × 10^−6^	4 × 10^−8^	0.9933	Non linear	0.8556	−5.7936	0.9850
4a/2	9 × 10^−7^	9 × 10^−8^	0.9841	Non linear	0.8151	−6.0323	0.9861
4c/2	1 × 10^−6^	9 × 10^−8^	0.9804	Non linear	0.9825	−5.9052	0.9847

^1^ The peak corresponds to the scan where the signal was observed for the first time.

**Table 3:** The performance characteristics of voltammetric methods developed for the quantitative determination of DIO.

Electrode	Technique	Linear Range (mol/L)	LoD (mol/L)	Sample	Ref.
CPE	SWV	-	2.66 × 10^−6^	-	[17]
CPE	SWV	1.25 × 10^−5^ to 2.00 × 10^−4^	2.66 × 10^−6^	human urine	[18]
GCE	AdSDPV	5.00 × 10^−8^ to 9.00 × 10^−6^	3.50 × 10^−8^	pharmaceuticals, serum	[19]
ErGONRs/GCE	SWV DPV	2.50 × 10^−8^ to 3.48× 10^−6^ 5.10 × 10^−8^ to 3.92 × 10^−6^	1.10 × 10^−8^ 1.50 × 10^−8^	pharmaceuticals	[20]
ZrO2-PDDA-Gr/GCE	DPV	5.00 × 10^−9^ to 2.00 × 10^−6^	2.00 × 10^−9^	fresh lemon leaves, tablets	[21]
PGE	DPV AdSDPV	1.00 × 10^−6^ to 1.00 × 10^−5^ 1.00 × 10^−7^ to 2.50 × 10^−6^	2.76 × 10^−7^ 7.42 × 10^−8^	dietary supplements	This work

CPE: carbon paste electrode; PDDA: poly(diallyldimethylammonium chloride); Gr: graphene; ErGONRs: electrochemical reduced graphene oxide nanoribbons; GCE: glassy carbon electrode; SWV: square wave voltammetry; AdSDPV: adsorptive stripping differential pulse voltammetry; DPV: differential pulse voltammetry.

**Table 4:** The repeatability values for the DIO voltammetry determination on PGE_H at different concentrations.

Technique	DPV			AdSDPV		
Concentration (mol/L)	1.00 × 10^−6^	5.00 × 10^−6^	1.00 × 10^−5^	1.00 × 10^−7^	5.00 × 10^−7^	2.50 × 10^−6^
RSD%_intraday_	5.72	3.21	1.96	4.84	3.91	2.21
RSD %_interday_	6.05	3.89	2.45	6.01	4.67	3.15

**Table 5:** The results obtained by the developed DPV method for quantitative determination of DIO in Revada dietary supplements.

Claimed DIO content (mg)	600
Found DIO content by DPV ± SD (mg)	599.22 ± 29.31
RSD, %	4.89
Average %R ± SD	99.87 ± 4.88
RSD, %	4.89
